# Successful surgıcal management of a giant neck teratoma in a newborn Baby: A case report

**DOI:** 10.1016/j.amsu.2022.104694

**Published:** 2022-09-15

**Authors:** Ismail Mohamed Ali, Mehmet Yaşar, Abdihakım Artan Abdi, Esın Seren Dırken

**Affiliations:** a Department of Otolaryngology–Head and Neck Surgery, Mogadishu Somalia Turkish Training and Research Hospital, Mogadishu, Somalia; b Department of Pathology, Mogadishu Somalia Turkish Training and Research Hospital, Mogadishu, Somalia

**Keywords:** Teratoma, Newborn, Neck mass, Magnetic resonance imaging, Head and neck teratomas

## Abstract

Neck tumors in newborns are very rare. Teratomas usually include all three germ cell layers, as well as tissues that are not native to the anatomic site of genesis. Teratomas of the head and neck make up a smaller percentage of congenital teratomas. Because of the external compression that oropharyngeal or neck masses produce, they can cause serious airway obstruction. In addition, the larynx or trachea may have an underlying lesion. We presented a mature, 1-day-old newborn with an isolated giant neck tumor and difficulty breathing. The intubation was successfully done and the entire mass was completely removed. Early neonatal life is explored to emphasize this challenge briefly with several interesting instances, including prenatal diagnosis, therapeutic alternatives, and ex-utero intrapartum therapy (EXIT) techniques.

## Highlights

• Teratoma should be evaluated as part of the differential diagnosis of neck masses, and confirmed or excluded before deciding on definitive therapy, despite its rarity.

## 1 Introduction

Fetal neck masses are uncommon, although they can be discovered during a fetal abnormality screening scan in the second trimester. It is critical to distinguish between the various diseases because they have an impact on prenatal counseling, antenatal care, and postnatal care [1]. Differentiating the various neck masses and obtaining an accurate diagnosis is critical for the prognosis and timing of surgical treatment in the neonatal period [1].

In children, teratomas are the most prevalent type of germ cell tumor [2]. The sacrococcygeal region, gonads, mediastinum, and pineal region are the most common sites of occurrence in children. Only 0.47%–6% of all teratomas are seen in the head and neck [2].

The ectoderm, mesoderm, and endoderm make up their histology. Airway obstruction is a common complication of neck teratomas [3]. In addition, newborns can have laryngeal or tracheal intrinsic lesions [3,4].

Teratomas of the head and neck can be highly vascularized, resulting in life-threatening blood loss [3]. To secure the embryonic airway, the EXIT technique is the preferable option.

Neck tumors in newborns are very rare. Teratomas of the head and neck make up a smaller percentage of congenital teratomas. We presented a mature, 1-day-old newborn with isolated giant neck tumor and difficulty breathing successfully managed with surgıcal excision.

## 2 Case report

**Fig. 1:**
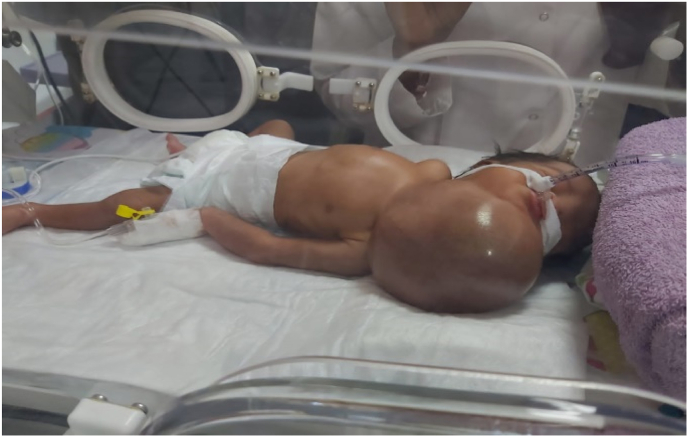
Clinical Image Showed Huge Neck mass with irregular lobulations.

A 1-day-old baby was brought to our hospital with a large neck mass and difficulty breathing. We urgently underwent endotracheal intubation due to difficulty breathing and were transferred to the neonatal intensive care unit. The mass was not discovered in utero, however, a polyhydramnios was mentioned during the gestational ultrasonography (US) scan. During the pregnancy, the mother did not consume any nicotine, alcohol, or medications. A large neck tumor was discovered during a physical examination (Fig. 1).

**Fig. 2:**
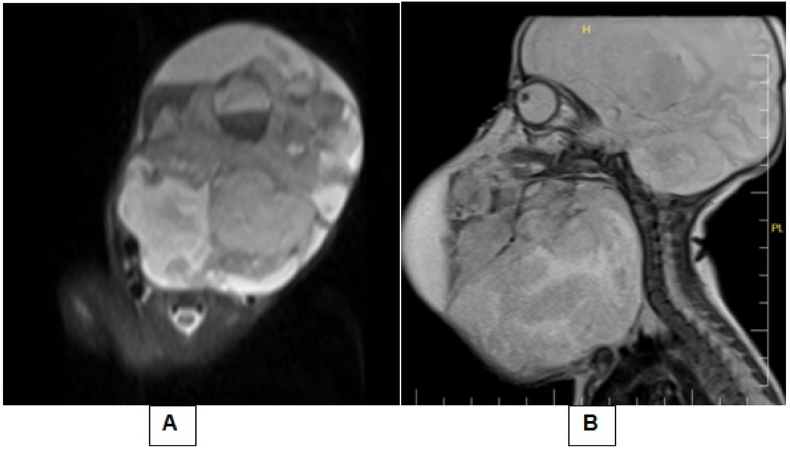
Axial T2 (A) and Sagital T1 (B) pre-contrast MRI scans show a massive well-defined multi-loculated complicated neck mass with considerable mass impact. The solid component has intermediate T2 and T1 signal intensities, whereas the cystic component exhibits high T2 and low T1 signal intensities, The esophagus and airway have been shifted.

Chest radiography, ultrasound of the abdomen, and echocardiogram were unremarkable. The tumor extended from the auricle to the clavicle on both sides of the neck. The US revealed a mass lesion containing hypoechoic, cystic areas of approximately 80 × 55mm in the middle and left side of the Neck. A neck Magnetic Resonance Imaging (MRI) was recommended and showed a massive well-defined multi-loculated complicated neck mass with considerable mass impact. The solid component has intermediate T2 and T1 signal intensities, whereas the cystic component exhibits high T2 and low T1 signal intensities. The esophagus and airway have been shifted (Fig. 2).

**Fig. 3:**
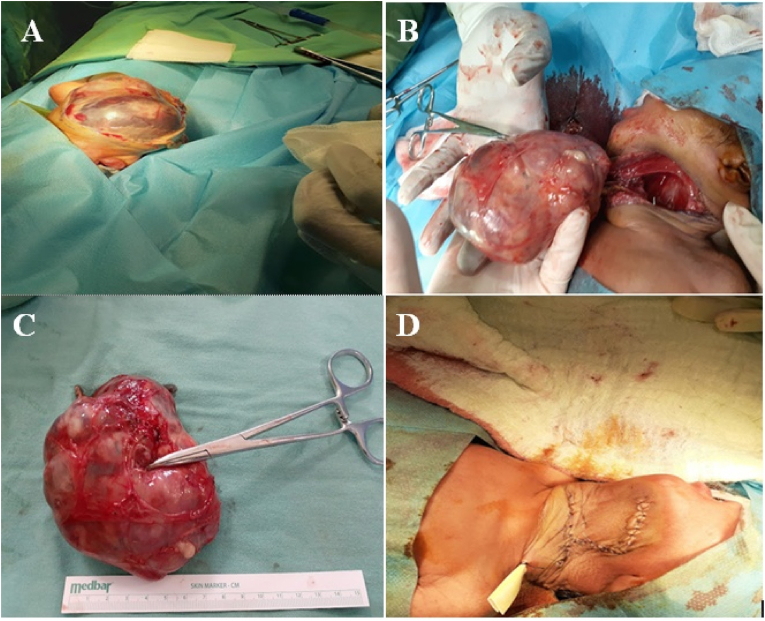
(A,B) The typical appearance of the lobular, larger soft tissue mass was captured during the surgical procedure, (C) Excised Specimen (D) Post Operative Skın Closure With Penrose Drainage. To avoid recurrence and malignant transformation, surgical management must be as complete as possible.

**Fig. 4:**
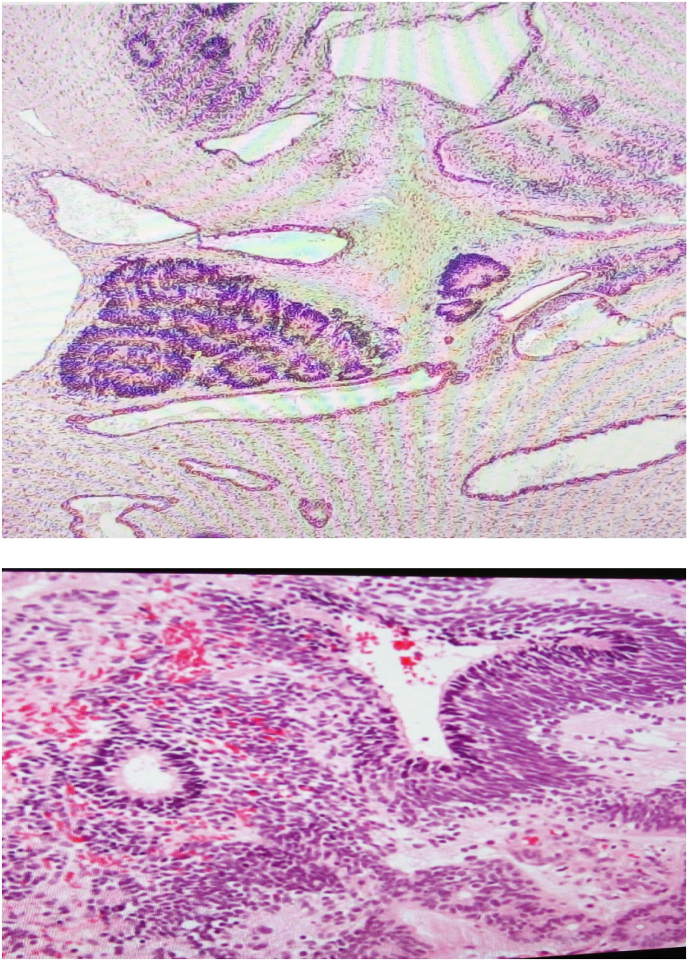
(A) areas of immature neural component with rosette formation in intermediate magnification, (B) Higher magnification of immature areas showing primitive morphology and focal rosette formation.

Once the baby has been stabilized in the neonatal intensive care unit, (Figure A) surgical intervention was undertaken. A left-sided incision was made over the tumor, and the tumor was securely attached to the tracheal cartilages in the midline. During the procedure, the tracheal cartilage was intact. The large mass was completely removed, and no other complications were encountered during the procedure (Fig. 3A,B,C,D). The entire procedure was uneventful. Immature Teratoma was the pathological outcome (Fig. 4A and B).

On postoperative day 7, a gastrostomy tube was used to keep the patient fed. On Day 45, the patient was discharged. During a one-year follow-up, the patient remained well, growing normally, and had no evidence of recurrence.

This work has been reported in line with the SCARE 2020 criteria [5].

## 3 Discussion

Most teratomas appear in the sacrococcygeal region, whereas, teratomas in the neck region comprise 2%–9% of all congenital teratomas [6,7]. Teratomas of the neck are reported in approximately 1:20,000 to 1:40,000 live births. Teratomas aren't usually cancerous. Despite this, they are associated with a high rate of morbidity and mortality [8,9]. Immature Teratoma was the pathological outcome of the current case.

The tumor compresses the oropharyngeal structures, causing airway obstruction in the fetus, which impairs swallowing and causes polyhydramnios. The tumor has the potential to displace and force the fetal lungs upward, resulting in severe lung hypoplasia, it's also one of the reasons for this newborn's death. Fortunately, our patient does not suffer from lung hypoplasia. The EXIT procedure is a surgical technique that permits a newborn to remain on placental support while the airway is secured [3].

Unfortunately, there was no accurate prenatal diagnosis in our baby, and the tumor was only identified by ultrasound after the mother underwent a cesarian section. As a consequence, our patient was unable to undergo an EXIT procedure due to post-delivery detection. Despite this, intubation was performed to secure the airway, and mechanical ventilation was initiated.

If the airway is secured prior to surgery, patients with cervical teratoma as an isolated anomaly can do well after postnatal excision. Patients with pulmonary hypoplasia are unable to be oxygenated, in addition to having a large cervical mass, and they die of respiratory insufficiency despite maintaining an adequate airway [1].

It's important to get a diagnosis as soon as possible when dealing with a newborn with a large lump in the neck region. Cystic hygroma, hemangioma, lipoma, dermoid cyst, congenital goiter, cervical meningocele, occipital encephalocele, bronchogenic cyst, branchial cleft cyst, thyroglossal duct cyst, and teratoma are all possible diagnosis for a congenital neck tumor [10,11].

## 4 Conclusion

Teratoma should be evaluated as part of the differential diagnosis of neck masses, and confirmed or excluded before deciding on definitive therapy, despite its rarity. Early neonatal life diagnosis is crucial to emphasize this challenge briefly with several interesting instances, including prenatal diagnosis, therapeutic alternatives, and ex-utero intrapartum therapy (EXIT) techniques.

## Ethical approval

According to our hospital rule, Ethical approval is only required in articles but not case reports.

## Sources of funding

There is no funding source for this study.

## Author contribution

All authors contributed toward writing, analysis, drafting, and revising the paper and they gave final approval of the version to be published, and agree to be accountable for all aspects of the work.

## Consent

The parent of the patient previously consented to use their medical and surgical data in this study. This study was carried out in accordance to the Helsinki Declaration contents.

## Registration of research studies

1. Name of the registry: Not applicable
2. Unique Identifying number or registration ID: Not applicable
3. Hyperlink to your specific registration (must be publicly accessible and will be checked): Not applicable

## Guarantor

Ismaıl Mohamed Ali.

## Provenance and peer review

Not commissioned, externally peer reviewed.

## Declaration of competing interest

The authors declare that there is no competing interest related to the study, authors, other individuals or organizations.
